# Telomere-to-telomere genome assembly of *Aspergillus nidulans* CS2498 with resolved centromeric regions

**DOI:** 10.1128/mra.00630-26

**Published:** 2026-09-14

**Authors:** Thomas Svoboda, Harald Berger, Joseph Strauss

**Affiliations:** 1 Department of Agricultural Sciences, Institute of Microbial Genetics, BOKU University Vienna 27270, Tulln an der Donau, Austria; University of California Riverside, Riverside, California, USA

**Keywords:** *Aspergillus nidulans*, telomere-to-telomere assembly, genome

## Abstract

*Aspergillus nidulans* is a well-characterized fungal model organism that is used to address basic genetic, epigenetic, and cell biological questions. Here, we present a gapless telomere-to-telomere assembly of nanopore sequencing reads of the eight chromosomes of *Aspergillus nidulans* CS2498, including centromeric regions.

## ANNOUNCEMENT

*Aspergillus nidulans* is a frequently used model organism to study basic and applied questions in molecular biology of simple eukaryotes. The genome of *A. nidulans* was first published in 2005 by Galagan et al. (1). Sequencing technology has advanced since those days, so this genome sequence lacks most parts of the telomeric and centromeric regions. These regions are, however, important to get a better understanding of chromosome biology and epigenetic regulation, including heterochromatin formation.

In this study, we completed the full genome of *A. nidulans*. DNA was isolated from strain CS2498 - veA1, pabaA1 (CS2498, Pontecorvo 1953 [2]), which is used as the wild-type reference strain in our lab. DNA was isolated according to the CENIS protocol (3), followed by sequencing using Oxford Nanopore MinION flow cell (Vienna Biocenter) with the following specifications: Model: dna_r10.4.1_e8.2_400bps_sup@v5.0.0; base calling: dorado Version 0.8.3; Kit: SQK-NBD114-96. For evaluation, default parameters were used except where otherwise noted. The reads with a total of 8.97 Gigabases (N50 = 5,136) were *de novo* assembled using Flye (version 2.5.9) (4) (flye-raw). The assembly was polished three rounds using RACON (version 1.5.0) (5), followed by evaluation of the assembly using BUSCO (version 6.0.0) (6), ascomycota_odb10. The evaluation returned a completeness of 99.5%. Further quality assessment using QUAST (version 5.2.0) (7) revealed a duplication ratio of 1.003 and a GC content of 50.13%, which is lower compared to the available reference genome (50.50%) due to the incorporation of the AT-rich centromeric regions in the actual sequence. In the next step, we employed the telomere-to-telomere toolkit quarTeT (version v 1.2.4) (8) for identification of centromeric and telomeric regions. QuarTeT teloexplorer (min_repeat_times 10) was used to identify the telomeric regions of the chromosomes (Table 1), while quarTeT centrominer was further used for centromere candidate prediction. We identified one large inversion of the size of around 400 kb in chromosome 5 compared to the reference genome of *A. nidulans* FGSC A4. Since we aimed to have the annotation consistent between the available reference genome and the new assembly, liftoff (version 1.6.3) (9) was used to alter the GFF file taking the available reference genome into account.

**TABLE 1:** Overview of chromosome length in the *de novo* assembly of the nanopore reads compared to the reference strain A4 and the number of telomer repeat monomers (AAGGTCT) identified at the end of the different chromosomes, as well as whole genome size indicated at the bottom

Chromosome	Chromosome length_CS2498	Chromosome length_A4	Telomere-repeats-left	Telomere-repeats-right
ChrI	3,758,513	3,704,807	14	12
ChrII	4,027,667	4,011,161	13	13
ChrIII	3,458,650	3,412,996	20	15
ChrIV	2,816,528	2,831,538	14	15
ChrV	3,208,243	3,145,033	18	15
ChrVI	3,392,247	3,354,444	12	10
ChrVII	4,551,670	4,492,219	19	11
ChrVIII	4,899,177	4,876,093	11	14
Genome	30,112,695	29,828,291		

## Data Availability

This Whole Genome Shotgun project has been deposited at DDBJ/ENA/GenBank under the accession JBZXTM000000000. The version described in this paper is version JBZXTM010000000. The genome is available on National Center for Biotechnology Information under project ID PRJNA1438182.
